# Health of Convicted Persons in the Third Generation of the Longitudinal Cambridge Study in Delinquent Development

**DOI:** 10.1177/0306624X211066837

**Published:** 2021-12-29

**Authors:** Guy C. M. Skinner, David P. Farrington

**Affiliations:** 1 University of Cambridge, UK

**Keywords:** Offending, Health, Injury, Hospital, Longitudinal Study

## Abstract

Research suggests that convicted persons are more likely than non-convicted persons to suffer poor health. However, few longitudinal studies have investigated associations between health and offending across generations. Using the Cambridge Study in Delinquent Development, this article prospectively investigates the relationship between health and offending across generations and between genders. At the average age of 25, third generation convicted males and females reported a higher incidence of serious drug use than non-convicted persons. Convicted males reported a higher incidence of mental illness and self-harm, whereas convicted females reported a lower incidence of physical illness, mental illness, self-harm and hospitalizations when compared to non-convicted females. Convicted males reported a higher incidence of industrial accidents, sports injuries and fight injuries, but a lower incidence of road accidents, whereas convicted females were more likely to report road accidents. Like their fathers, convicted males show worse health compared to non-convicted individuals.

## Background

To date, research has provided considerable evidence for the presence of health inequalities between people with different social backgrounds and/or roles within the social system. For example, research has shown that wealth, education, and employment increase differences in physical and mental health outcomes observed between these individuals (Adler & Newman, 2002; Amone-P’Olak et al., 2009; Quon & McGrath, 2015; Schreier & Chen, 2013; Skalamera & Hummer, 2016). However, research into the impact of criminality on health has been limited in scope, with academic interest only beginning to emerge recently (Jackson & Vaughn, 2018; Tibbetts, 2014).

Current literature indicates that risks of physical health problems are significantly higher among individuals with convictions compared to those with no convictions (Binswanger et al., 2009; Cloud, 2014; National Commission on Correctional Health Care, 2002; Skinner et al., 2020). Outside of incarcerated populations, there is evidence that juvenile and adult individuals with criminal justice involvement are at increased risk of injury in general (Lewis & Shanok, 1977). Earlier literature also suggested that sexually transmitted and blood-borne infections are more common among individuals with criminal justice involvement (Edwards et al., 1999; Ruiz et al., 1999). In addition, tuberculosis, respiratory illness, and epilepsy (Feinstein et al., 1998) are also frequently seen among convicted persons.

It has also been evidenced that there is a significant difference in the rates of mental health and substance use problems between young people who have had encounters with the criminal justice system as compared to those who have had no contact (Chassin, 2008; Kessler et al., 2005; McReynolds et al., 2010; Shufelt & Cocozza, 2006). The actual level of disparity may differ (Wasserman et al., 2010), but an increased rate of between 40% and 70% having mental health problems and substance abuse issues has been found (Abram et al., 2015; Fazel et al., 2008; Teplin et al., 2002). This is in contrast to rates nearer 10% to 20% found amongst those who do not encounter the courts while young (Merikangas et al., 2010; SAMHSA, 2014; Wu et al., 2011). Additionally, among the most common predictors of reoffending are substance abuse (Bennett et al., 2008; Hoeve et al., 2013; Schubert et al., 2011) and comorbid substance difficulties together with mental health problems (Chassin, 2008; McReynolds et al., 2010).

Research has indicated that depression, anxiety, and post-traumatic stress disorders are common amongst young people who have contact with the criminal justice system (Karnik et al., 2009). This finding has been reinforced by a recent meta-analysis, establishing that, while both externalizing mental disorders and comorbid externalizing/internalizing disorders had links to criminal behavior, internalizing problems did not reveal such an association (Wibbelink et al., 2017).

Researchers have also argued that the negative effects of imprisonment on health can occur in a variety of ways (Braverman & Murray, 2011; Pajer et al., 2007). These include that being in prison risks exposure to infection and disease (Cloud, 2014; Johnson & Raphael, 2009; Massoglia, 2008a, 2008b; National Commission on Correctional Health Care, 2002; Thomas & Torrone, 2008). Studies show that stress factors, created by imprisonment, can exacerbate pre-existing health concerns (Desai et al., 2006; Holman & Ziedenberg, 2006; Wasserman & McReynolds, 2011). The actual act of being incarcerated itself can also generate life-long stress effects which, in turn, can have deleterious health consequences (Massoglia, 2008a). Further research highlights that being a former convict has impacts on both family and job opportunities, and in turn this creates health concerns (Massoglia, 2008b; Ross et al., 1990). There is also evidence that imprisonment can lead to mental health problems (Fazel & Seewald, 2012; Fazel et al., 2016; Schnittker et al., 2012).

Impacts on the health of those who are related to convicted persons have also been found (Turney, 2014a; Turney & Haskins, Forthcoming; Turney, 2014b; Turney et al., 2012; Turney, 2017a, 2017b; Turney & Goodsell, 2018; Turney & Wildeman, 2013, 2015a, 2015b; Wakefield & Wildeman, 2014; Wildeman et al., 2018). For example, Turney (2014b) found speech and developmental problems amongst the children of previously incarcerated parents. Jackson and Vaughn (2017a) showed that children of convicted individuals experienced poor sleep and had eating concerns compared with children of non-incarcerated parents. Overall, there are clear familial consequences of offending in terms of poor health, especially if the parent was previously incarcerated (Wildeman et al., 2018; for reviews, see Foster & Hagan, 2015; Haskins & Turney, 2017; Murray et al., 2012, 2014; Turney & Goodsell, 2018).

Many theories have been proposed within Criminology to explain the relationship between offending and health (Vaughn et al., 2020). For example, Gottfredson and Hirschi’s (1990) General Theory of Crime, which links crime to low self-control, can provide useful insights into the health and crime relationship. This theory has spawned investigations into the links that may exist between low self-control and a wide range of undesirable behaviors (Arneklev et al., 1993; Evans et al., 1997; Paternoster & Brame, 1998; Pratt & Cullen, 2000).

A general outcome of these works is the postulate that those in the population with low self-control require immediate satisfaction of their impulses, and that they tend to achieve that satisfaction without due assessment of the future consequences for their life chances or future physical wellbeing. Gottfredson and Hirschi (1990) themselves go further and argue that offenders generally risked worse health in their future years than non-offenders, stating that “. . .offenders tend to be involved in accidents, illness, and death at higher rates than the general population” (p. 94). Additionally, Gottfredson and Hirschi (1990) argue that “. . .the traits composing low self-control are also not conducive to the achievement of long-term individual goals. On the contrary, they impede educational and occupational achievement, destroy interpersonal relations, and undermine physical health . . . well-being” (p. 96). Thus, this theory argues that individual differences in self-control influence poor physical health and early mortality.

This theoretical link has not been extensively explored by subsequent researchers. Where it has been studied, the consensus is that low self-control is indeed a predictor of premature mortality. The two main studies of this type linked low self-control to early death, especially from homicide (Piquero et al., 2005), and showed that poor choices led to lower wellbeing and worse health problems (although the correlation was not very strong; see Miller et al., 2011; Barnes et al., 2011 ).

Moffitt’s (1993) developmental taxonomy is another theory that can shed light on the relationship between health and offending. Her theory, latterly extended, proposes that there are three types of offenders, each of which has a different etiology, behavior and outcomes that have impacts throughout the lives of types of convicted persons. The first of these types she termed “adolescence-limited.” Here, early offending is related to peer influence and to the strain between fast-growing physical capability and desires and low social access to adult behaviors (McGee & Moffitt, 2019). Consequently, rule-breaking in areas such as vandalism, drug taking, and delinquency occurs. However, these undesirable behaviors are time-limited and largely cease as adulthood proper is reached.

The second category Moffitt termed “life-course-persistent” (LCP). In this group a far more long-term and definable etiology is seen to exist. This starts with the in-utero effects of maternal actions and continues with a childhood environment that does not have the ability to address and remedy the inherent individual problems that increasingly develop (McGloin et al., 2006). For example, evidence suggests that LCPs are influenced by cognitive deficits, an under-controlled temperament, hyperactivity, poor parenting, poverty, disrupted families, genetic and biological factors (for a review see McGee & Moffitt, 2019). Such childhood disadvantage is then reinforced once more by poor opportunities in adolescence, and these restrictions are accompanied by antisocial and ultimately criminal behaviors. These entrenched characteristics, Moffitt argues, continue across the whole of adult life, during which chances to change path are infrequent.

Only a limited amount of research has linked this taxonomy to mortality or poor health, with the majority of studies choosing to purely examine antisocial and criminal elements. This is despite Moffitt predicting that “life-course-persistent’ antisocial lifestyles, violence, socioeconomic stress, and hostile personality will place them at greatest risk in midlife for poor physical health, cardiovascular disease, and early mortality” (Moffitt, 2003, p. 65).

Moffitt’s theorizations have been tested and confirmed by other authors. For example, Piquero, Daigle et al.(2007) investigated poor well-being in the context of Moffitt’s taxonomy, analyzing the Baltimore part of the National Collaborative Perinatal Project, which was a longitudinal study of several thousand subjects who were followed from birth to ages 27 to 33. Comparing adolescence-limited to long-term offenders, this work showed that it was the latter who were more likely to experience physical health issues. Additionally, the latter group also adopted antisocial practices (e.g., alcohol, cigarette, and drug use), increasing their likelihood of later poor health (Piquero et al., 2007b).

Piquero et al. (2010, 2011), Skinner et al. (2020) and Skinner et al. (2021) have related offending to physical health, mental health, hospitalizations and early death in the Cambridge Study in Delinquent Development (CSDD). They found that, by age 48, offending trajectories differentially predicted health outcomes, with high-rate chronic offenders at the greatest risk—even when logistic regression modeling ruled out individual or environmental childhood risk factors for offending as a likely common cause of offending and health problems (Piquero et al., 2014).

In two further studies using data that examined trajectories in the Dunedin Multidisciplinary Health and Human Development Study, Odgers et al. (2007) firstly found at age 32 that, of four conduct problem trajectories (childhood-onset/life-course-persistent, adolescent-onset, childhood-limited, and low), the first cluster experienced the worst health burden—in terms of mental and physical health problems at 32 years of age measured via diagnostic interviews and physical examinations. In the second study using the same source of data, these researchers found a link between the use of alcohol or drugs before age 15 and an elevated likelihood of early pregnancy, school failure, substance dependency, sexually transmitted diseases, and criminal activities. These results held up independently of early childhood behavior (Odgers et al., 2008).

The crucial difference between Gottfredson and Hirschi (1990) and Moffitt (1993) is that the former would argue that life-course-persistent offenders differ in degree from other offenders, whereas the latter would argue that these two types of offenders differ in kind, because they are influenced by different risk factors. However, both theories would predict that life-course-persistent offenders are more extreme than other offenders in their deviant lifestyles. To the extent that poor physical health and early deaths are caused by deviant, risky and unhealthy lifestyles, it would be expected that life-course-persistent offenders would, on average, have worse physical health and die earlier than other offenders. Both theories would predict that the prevalence of physical health problems and early death would increase from non-offenders to one-time offenders to recidivists and then to chronic offenders (defined as committing five or more offenses; see Farrington, 2020). However, while Gottfredson and Hirschi (1990) would predict that adolescence-limited offenders would tend to die earlier than non-offenders, we would argue that Moffitt (1993) would not predict this, because adolescence-limited offenders would become similar to non-offenders after they give up offending. Indeed, Farrington et al. (2006) found that “desisters,” who were convicted up to age 20 but not subsequently, were very similar to non-offenders in life success measures at age 48.

A third life-course criminology theory which may be useful in informing our hypotheses, and explaining the health versus crime relationship, is Sampson and Laub’s (1993) Age-Graded Theory of Informal Social Control. Sampson and Laub’s theory has similarities with Moffitt, in that it stresses life trajectories and divergent pathways. However, their age-graded theory of informal social control developed from the work of Elder (1995, 1998) and emphasizes the importance of two distinct, yet cooccurring phenomena: state dependence and population heterogeneity. Future behavior, according to Sampson and Laub, is a reflection of (a) the social consequences of previous interactions and behaviors, as well as (b) individual differences in the predisposition for committing that behavior.

To provide an example, Sampson and Laub acknowledge that delinquency and social ties (to conventional individuals and institutions) have a reciprocal relationship over the life course, so that early bonds reduce the risk of adolescent offending, but that such offending (along with a criminal record) will also conflict with potential adult bonds, such as marriage and work. As a result, the mutual reinforcement of attenuated social bonds and delinquency over time is a potent explanation of stability in behavior. Nonetheless, to account for change, they also accommodate serendipitous life occurrences that can act as tipping points, steering individuals away from a criminal career. For instance, life transitions such as marriage or stable employment may operate as mechanisms of informal social control, situating individuals within the confines of a law-abiding life. Furthermore, human agency and luck, according to Sampson and Laub (2003), also contribute to criminal desistance.

Therefore, this theory is an age-graded theory of informal social control in that diﬀerent sources of informal control have the potential to be turning points at diﬀerent stages during the life course. This theory has several studies providing evidence to support its theorizations (Doherty, 2006; Sampson et al., 2006; Wright & Cullen, 2004). Indeed, studies have found significant evidence of health differences among people with differing social identities and/or positions in the social structure. Inequalities in wages, schooling, and housing, for example, have been shown to increase differences in physical health (Adler & Newman, 2002; Amone-P’Olak et al., 2009; Quon & McGrath, 2015; Schreier & Chen, 2013; Skalamera & Hummer, 2016).

The theories of Gottfredson and Hirschi (1990), Moffitt (1993) and Sampson and Laub (1993) are informative in explaining both individual and group health and behavioral patterns. However, there remains a paucity of longitudinal and non-incarceration focused investigations of the associations between offending and health outcomes (Laub & Vaillant, 2000; Odgers et al., 2007; Piquero et al., 2010; Piquero, Farrington et al., 2007; Samuelson et al., 2010; Testa & Semenza, 2020). Indeed, in their review of criminal career research, Piquero, Farrington et al. (2007, p. 211) state that “although researchers have long established a linkage between offending and poor health outcomes, little specific information is known.”

To date, most research has utilized prison samples, drawing conclusions which are oriented toward this subgroup of individuals with criminal justice system involvement. In this regard, important new opportunities have emerged in the Cambridge Study in Delinquent Development (CSDD) which has collected prospective longitudinal data on injury, illness and treatment-seeking behavior of 411 London males who were first assessed at age 8-9 in 1961-62. These males are termed generation 2 (G2). Their parents are generation 1 (G1) and their children are generation 3 (G3).

Prior research investigating the relationship between health and offending in the CSDD, in G2 males at ages 18, 32, and 48 (Shepherd et al., 2002, 2004, 2009) has found both positive and negative associations. In general, convicted persons, compared with non-convicted persons, were found to be healthier at age 18, but relatively unhealthy by age 48. More specifically, convictions were found to be associated with fewer respiratory illnesses and fewer illnesses overall at age 18 and fewer organic illnesses at age 32. Furthermore, concurrent antisocial behavior was inversely related to respiratory infections at age 18 and to hospital admissions at age 32, possibly because of a protective effect of alcohol against infection (Cohen et al., 1993). Childhood precursors of offending which were linked to a lower risk of infections at age 18 were high daring and low income. However, poor parental supervision predicted a higher risk of cardiovascular illness at age 32, while low nonverbal IQ predicted a higher risk of psychological illness at the same age (Shepherd et al., 2002, 2004, 2009).

Further inverse relations were found between antisocial behavior at age 18 and health outcomes at age 32, principally between heavy alcohol consumption and infections and organic illness. Self-reported offending at age 32 was related to low hospital admissions at the same age, although this may be because offenders were less likely to seek treatment (Shepherd et al., 2002, 2004). On the other hand, by age 32 earlier antisocial behaviors such as fighting after drinking and heavy smoking were positively related to illness (Cohen et al., 1993), particularly mental illness, and low job status was positively related to hospital admissions. Overall, a consistent finding up to age 32 in the CSDD was the link between childhood precursors of antisocial behavior and later injury, convictions and concurrent antisocial behavior. By age 48, convicted persons had a higher incidence of mental illness, and, when adjusting odds ratios for common risk factors for offending (Farrington et al., 2015), also had a higher incidence of hospitalizations but a lower incidence of disabling medical conditions (Shepherd et al., 2009).

Furthermore, little research has investigated intergenerational patterns of health in justice involved populations. This is surprising, given that a predisposition toward criminogenic behaviors has been found to be partly heritable (Barnes et al., 2011; Chabris et al., 2014; Polderman et al., 2015; Turkheimer, 2000), and the recent debates on “nature and nurture” in criminology and the contributions of biosocial research (Burt & Simons, 2014). Indeed, while the number of significant findings from biosocial research are far too numerous to discuss here (see Barnes et al., 2016; Beaver et al., 2015; Belsky & Pluess, 2009; Raine, 2013; Walsh & Beaver, 2009, for summaries), the growing body of evidence revealing genetic influences on criminal/antisocial behavior has reached a “critical mass” (Burt, 2009a, 2009b; Ferguson, 2010; Mason & Frick, 1994; Miles & Carey, 1997; Rhee & Waldman, 2002), “. . . indicating that the question of whether genes matter is no longer a question at all; they do” (Barnes et al., 2014, p. 472).

There are also high levels of intergenerational transmission of convictions (Farrington et al., 2017) between offenders and their children, which makes it seem likely that the associated negative effects of offending on physical health would also be present in their children. Research to date has mainly focused on investigating the impact of family members being incarcerated on a child (Jackson & Vaughn, 2017; Murray et al., 2014; Turney, 2014; Wildeman et al., 2018), leaving a significant area of inquiry yet untouched.

New data in the CSDD has allowed us to examine continued associations between health and offending, building upon the aforementioned findings from the G2 males (Skinner et al., 2020). To our knowledge, no studies have investigated the relationship between offending, physical health, self-harm, mental health and hospitalization records of a third generation of individuals in a longitudinal study and few that have investigated both male and female convicted persons (Piquero et al., 2007; Samuelson et al., 2010; Testa & Semenza, 2020). Of the few studies that have investigated gender differences in the relationship between offending and health, mixed results are reported. For example, Samuelson et al. (2010) observed no differences between genders, whereas Testa and Semenza (2020), drawing on data from four waves of the National Longitudinal Study of Adolescent to Adult Health (*N* = 9037), observed that males in their sample engaged in offending at persistently higher rates than females, and tended to report worse self-rated health. However, males also reported fewer depressive symptoms on average. The present article, informed by previous theoretical work, reports the first analyses of the relationship between convictions and health of a third generation of individuals, and directly compares them to their G2 fathers.

## Research Questions

The main aim of this study is to investigate to what extent differences in health between convicted and unconvicted G2 males are replicated in their children (G3 males and G3 females). It is important to investigate the extent to which relationships between health and convictions are replicated across generations. This provides us insight into possible time points, environments and relationships which affect health in the context of crime, in addition to any gender differences that may exist. Subsequently, this information may further inform the mechanisms of successful intervention which seek to mitigate intergenerational impacts of both convictions and poor health outcomes.

## Hypotheses

Since the G2 fathers were selected because they were living in a deprived area (and their own G1 fathers overwhelmingly had low status manual jobs), we expect that the G3 children will have fewer social problems, because of regression to the mean. Furthermore, due to a pattern of upward mobility in the G2 fathers, transitioning from manual labor-based work to middle class jobs and the environmental change in neighborhood this facilitated, we expected that fewer of the G3 sons would have convictions, and fewer would have health problems. Our main hypothesis is that differences in health between convicted and unconvicted G3 males will be similar to corresponding differences for G2 males. We also expect some similarities in results between G2 males and G3 females, and between G3 males and G3 females, but we cannot propose firm hypotheses in these cases because of the paucity of prior knowledge.

## Method

### The CSDD

The CSDD began in 1961, and for the first 20 years it was directed by Donald West. David Farrington started working on it in 1969, and began directing the CSDD in 1982. The most recent data collections were jointly directed by David Farrington and Jeremy Coid. The results of the CSDD have been described in six books (Farrington et al., 2013; Piquero, Daigle et al., 2007; West, 1969, 1982; West & Farrington, 1973, 1977), and in eight summary articles (Farrington, 1995b; Farrington et al., 2021; Farrington, 2003, 2019; Farrington, Coid, & West, 2009; Farrington, 2021; Farrington & West, 1981, 1990).

At the time they were first contacted in 1961to 62, the G2 boys were all living in a working-class area of South London. The vast majority of the sample was chosen by taking all the boys who were then aged 8 to 9 and on the registers of six state primary schools within a one-mile radius of a research office which had been established. In addition to 399 boys from these six schools, 12 boys from a local school for “educationally subnormal” children were included in the sample, in an attempt to make it more representative of the population of boys living in the area. Therefore, the G2 boys were not a probability sample drawn from a population, but rather a complete population of boys of that age in that area at that time. The males have been interviewed or assessed nine times, at ages 8, 10, 14, 16, 18, 21, 25, 32, and 48. Most of the G2 boys were born in 1953.

Most of the G2 boys (357, or 87%) were White in appearance and of British origin, in the sense that they were being brought up by parents who had themselves been brought up in England, Scotland, or Wales. Of the remaining 54 boys, 12 were African-Caribbean, having at least one parent of West Indian (usually) or African origin. Of the remaining 42 boys, 14 had at least one parent from the North or South of Ireland, 12 had parents from Cyprus, and the other 16 boys had at least one parent from another Western industrialized country.

On the basis of their fathers’ occupations when they were aged 8, 94% of the G2 boys could be described as working-class (categories III, IV or V on the Registrar General’s scale, describing skilled, semi-skilled or unskilled manual workers), in comparison with the national figure of 78% at that time. The majority of the boys were living in conventional two-parent families with both a father and a mother figure; at age 8, only 6% of the boys had no operative father and only 1% had no operative mother. This was, therefore, overwhelmingly a traditional White, urban, working class sample of British origin.

### Interviews

As mentioned, the G2 males have been assessed or interviewed nine times, at ages 8, 10, 14, 16, 18, 21, 25, 32, and 48. At all ages except 21 and 25, the aim was to interview all the males who were still alive, and it was always possible to interview a high proportion: 405 (99%) at age 14, 399 (97%) at age 16, 389 (95%) at age 18, 378 (94%) at age 32, and 365 (93%) at age 48.

Between 2004 and 2013, efforts were made to interview the biological G3 children of the G2 males. There were 691 G3 children whose name and date of birth were known. Only children aged at least 18 (born up to 1995) were targeted. The ethical requirements of the South-East Region Medical Ethics Committee required that the G2 male and/or his female partner were contacted in trying to interview the G3 children. Therefore, 20 children whose fathers refused at age 48, and 7 children who father was dead at age 48 (and where no female partner was available) were not eligible to be interviewed. An additional six G3 children who had died and three who were disabled (one Down’s syndrome, one mental health problems, one severe attention deficit-hyperactivity disorder), together with two who did not know that the G2 male was their father, were also considered to be not eligible.

Of the 653 eligible G3 children, 551 were interviewed (84.4%); 291 of the 343 G3 males (84.8%) and 260 of the 310 G3 females (83.9%). Of the remainder, 39 children refused, 33 parents refused, 13 children could not be traced, 14 were elusive (agreeing or not refusing but never being available to interview), and three were aggressive or problematic. This article focuses on the G3 children. They were interviewed at the average age of 25 and asked to report illnesses, injuries, accidents and hospitalizations in the previous 5 years. Interviews typically took 1.5 hours to complete, and were predominantly conducted face-to-face in private within the G3’s homes. The interview was conducted by trained researchers and was determined to meet the ethical standards of the South East Region Medical Ethics Committee.

### Criminal Record Searches

The minimum age of criminal responsibility in England and Wales is 10. Officially recorded cautions were counted as well as convictions in the Police National Computer (PNC), since cautions were routinely recorded on a national basis from 1995. In this article, “convictions” include cautions, which can be given to adults as well as juveniles and both of which require proof that the person has committed the crime. Cautions are typically given for younger offenders and less serious crimes. Both convictions and cautions are more serious than arrests because arrests may not necessarily lead to a finding of guilt, which is required for both convictions and cautions.

Over time, there has been a tendency to replace convictions with cautions, so many cautions are given nowadays where there would have been convictions in days gone by. Convictions were only counted if they were for “standard list” (more serious) offenses, thereby excluding minor crimes such as minor traffic infractions and simple drunkenness. The most common offenses that were included were thefts, burglaries and unauthorized takings of vehicles, although there were also quite a few offenses of violence, vandalism, fraud and drug abuse. The definition of what is a “standard list” offense changed over time. In particular, common assault became a standard list offense in July 1995, drunk driving was added to the standard list from January 1996, and being drunk and disorderly was added in April 1997. All of these types of offenses were counted.

As mentioned, there were 691 G3 children whose name and date of birth were known. Their median year of birth was 1981, and more than half were born between 1977 and 1985. They were first searched in microfiche records in 1994, and they were then searched in the PNC in 2003, 2006, 2011 to 12, and most recently in 2017. We have always counted the age when an offense was committed, not the age at the time of a conviction. In light of these considerations, the median age at which the G3 children were last searched for their offenses was 34, and more than half were last searched between ages 30 and 37. This median age is 34 and not 36 because of delays between the date that the offense was committed, the date of conviction, and the date that the conviction was entered in the PNC. The 31 G3 children who were abroad could not be searched, but 655 of the remaining 660 were considered to be searched. These included 342 G3 males and 313 G3 females. The other five cases had common names, and it was not clear that the correct person had been searched. Almost all G3 children (92%) were searched at least up to age 25.

During their interview at the average age of 25, the G3 children were also asked to admit how many times they had committed specified offenses in the previous 5 years. These offenses included burglary, theft of vehicles, theft from vehicles, shoplifting, theft from slot machines, theft from work, vandalism, assault (defined according to involvement in fights), starting fights, and driving after drinking at least 10 units of alcohol, which would have caused them to fail the breathalyzer test. (1 unit = 10 ml. of pure alcohol. This is approximately a half-pint of beer or cider, a small glass of wine, or a single measure of spirits.) They were given a score of how many offenses they admitted out of 10.

### Health Data

The illnesses reported by the G3 children were categorized into Physical Illnesses, Mental Illnesses and Hospitalizations. Comparisons were made between those with convictions and those without convictions. The main question was “Have you had any illnesses or injuries in the past 5 years that have made you unfit for work or education for a week or more?” Information on self-harm and drug use was also available, defined here as cases of self-harm and drug use which resulted in medical treatment of any kind. Injuries were categorized according to their cause as sport injuries, assault injuries, industrial injuries, and road accidents. These questions were open-ended and relied upon self-reports. There were 530 G3s with full health and offending data, 275 males and 255 females (see Table 1). Comparable data were available for G2 males at age 32, which is also presented in Table 1 for comparison. As expected, more of the G2 males were convicted than of the G3 males (43.9% compared with 29.5%), and more of the G3 males were convicted than of the G3 females (29.5% compared with 11.0%).

**Table 1.** G3 and G2 Frequencies of Offenders and Non-Offenders.

	G3 individuals (%)	G3 male (%)	G3 female (%)	G2 male (%)
Non-offenders (NO)	421 (79.4)	194 (70.5)	227 (89.0)	220 (56.1)
Offenders (OFF)	109 (20.6)	81 (29.5)	28 (11.0)	172 (43.9)
Total	530 (100.0)	275 (51.9)	255 (48.1)	392 (100.0)

## Analysis

In light of the relationship between age searched and the probability of conviction, the relationship between health and offending for G3 children was investigated by only using convictions recorded up to age 25 for G2s, thereby controlling for age differences. Where the incidence was 0 in the cell of a 2 × 2 table, Haldane-Anscombe corrections were used. This is a common practice that involves adding 0.5 to each of the empty cells and then calculating the Odds Ratio (*OR*) using these adjusted cell counts (Agresti & Min, 2002; Ruxton & Neuhäuser, 2013). This also removes some bias from the estimator, and is the default adjustment in multiple statistical packages for any occurring empty cells in a 2 × 2 table.

Because the G3 children are not all independent, we adjusted the variance of effect sizes (logarithms of odds ratios) to take account of the clustering of G3 children in G2 families. For convictions of G3 males, the Intraclass Correlation (ICC) was .27, implying that the Standard Error (*SE*) should be increased by 7.1%. For convictions of G3 females, the ICC was .08, implying that the *SE* should be increased by 2.0%. It is not surprising that the clustering of convictions in families was greater for G3 males, because convictions of G3 females were much less prevalent. We therefore increased the standard errors of G3 Males by 7.1% and the standard errors of G3 Females by 2.0%, in line with previous work on this sample (see Farrington & Jolliffe, 2021). Data were analyzed by means of the Statistical Package for the Social Sciences version 24.

## Results

Table 2 presents the ORs for health of G3 convicted persons compared to non-convicted persons. Comparable data for G2 males are also shown. G3 convicted males had a higher incidence of mental illness, self-harm and drug use compared to non-offenders. G3 convicted females had a higher incidence of drug use than non-convicted females, but had a lower incidence of physical illness, mental illness, self-harm and hospitalizations. Like the G3 males, the G2 males had a higher incidence of mental illness and a very high OR for drug use. The ORs of the G2 males were remarkably similar to the ORs of the G3 males, showing impressive replication over two generations of males.

**Table 2.** Incidence and Odds Ratios for Health of G3 and G2 Offenders Compared to Non-Offenders.

	Physical illness		Mental illness		Ever hospitalized		Drug use		Self-Harm	
	*I*	*OR*	*I*	*OR*	*I*	*OR*	*I*	*OR*	*I*	*OR*
OFF(G3M)	15 (18.5)	1.16 (1.14–1.80)	6 (7.4)	2.39* (1.58–3.58)	4 (4.94)	0.80 (0.65–1.01)	11 (13.6)	6.48* (4.03–10.12)	9 (11.1)	2.71* (1.97–3.87)
NO(G3M)	32 (16.5)	—	6 (3.1)	—	14 (7.2)	—	4 (2.1)	—	8 (4.1)	—
OFF(G3F)	5 (17.6)	0.67* (0.66–0.99)	1 (3.6)	0.39* (0.25–0.63)	2 (7.1)	0.67* (0.52–0.87)	3 (10.7)	2.78* (2.15–3.59)	2 (7.1)	0.63* (0.50–0.84)
NO(G3F)	61 (26.9)	—	14 (6.2)	—	21 (9.3)	—	3 (1.3)	—	26 (11.5)	—
OFF(G2M)	37 (21.5)	0.93 (.67–1.16)	9 (5.2)	5.20* (2.66–10.10)	27 (15.7)	1.11 (.87–1.42)	5 (2.9)	6.40 (0.74–55.25)	2 (1.2)	2.56 (0.23–28.45)
NO(G2M)	51 (23.2)	—	2 (1.00)	—	28 (12.7)	—	1 (0.45)	—	1 (0.45)	—

*Note*. I = incidence, %’s in parentheses; OFF(G3M) = generation 3 male offenders; NO(G3M) = generation 3 male non-offenders; OFF(G3F) = generation 3 female offenders; NO(G3F) = generation 3 female non-offenders; OFF(G2M) = generation 2 male offenders; NO(G2M) = generation 2 male non-offenders; OR = odds ratio.

* *p* < .05.

Table 3 presents the *OR*s of injuries of G3 convicted individuals compared to non-convicted persons. G3 convicted males had a higher incidence of industrial accidents, sports injuries and fight injuries, but a lower incidence of road accidents. G3 convicted females, however, had a higher incidence of road accidents compared to non-convicted females. Like the G3 males, the G2 convicted males had a higher incidence of fight injuries, but otherwise their results were rather different from those of the G3 males.

**Table 3.** Incidence and Odds Ratios for Injuries of G3 and G2 Offenders Compared to Non-Offenders.

	Road accidents		Industrial accidents		Sports injuries		Fight injuries	
	*I*	*OR*	*I*	*OR*	*I*	*OR*	*I*	*OR*
OFF(G3M)	1 (1.2)	0.33* (0.12–0.60)	10 (12.3)	1.98* (1.50–2.63)	10 (12.3)	1.98* (1.45–2.43)	4 (4.9)	2.33* (1.41–3.62)
NO(G3M)	9 (4.6)	—	12 (6.2)	—	16 (8.2)	—	4 (2.1)	—
OFF(G3F)	3 (10.7)	2.43* (1.71–3.44)	0 (0.0)	0.73 (0.54–10.10)	0 (0.0)	0.61 (0.43–10.09)	0 (0.0)	0.89 (0.40–11.21)
NO(G3F)	10 (4.5)	—	5 (2.3)	—	6 (2.7)	—	4 (1.8)	—
OFF(G2M)	9 (5.2)	1.64 (0.60–4.50)	18 (10.5)	0.95 (.74–1.21)	12 (7.0)	1.15 (.84–1.63)	22 (12.8)	2.35* (1.13–4.87)
NO(G2M)	7 (3.2)	—	24 (11.1)	—	12 (5.5)	—	12 (5.5)	—

*Note*. I = incidence, %’s in parentheses; OFF(G3M) = generation 3 male offenders; NO(G3M) = generation 3 male non-offenders; OFF(G3F) = generation 3 female offenders; NO(G3F) = generation 3 female non-offenders; OFF(G3M) = generation 2 male offenders; NO(G2M) = generation 2 male non-offenders; OR = odds ratio.

* *p* < .05.

There is a possibility of Type I error (rejecting the null hypothesis when it is true) in these multiple analyses. Some researchers have used the Bonferroni *p*-value correction to deal with this problem. However, this correction has been strongly criticized, for example because it increases Type II error (failing to reject the null hypothesis when it is false) and therefore decreases statistical power (e.g., Feise, 2002; Nakagawa, 2004; Perneger, 1998). It seems preferable to compare the fraction of tests that are statistically significant with the chance expectation (based on *p* = .05) of 1 in 20. For example, in Table 2, 9 out of 15 tests were statistically significant at *p* = .05, far greater than the chance expectation of 1 in 20. In Table 3, 6 out of 12 tests were statistically significant, again far greater than the chance expectation of 1 in 20. Because of small numbers, large effect sizes are sometimes non-significant. The numbers were small because of the very severe criteria for counting illnesses, injuries and drug use.

## Discussion

Offending is part of a constellation of social disorders and is widely considered to be a public health issue (Kinner & Young, 2018; Macdonald, 2002; Shepherd & Farrington, 1993), with research suggesting that offending may in some way influence poor health and premature mortality (Farrington, 1995a; Junger et al., 2001; Piquero et al., 2011; Shepherd et al., 2002; Skinner & Farrington, 2020a; Skinner et al., 2020; Skinner & Farrington, 2020b).

This study investigated the associations between health, injuries and offending in the third generation of the CSDD, at the average age of 25. Our findings reinforce previous research which identifies associations between offending and poor health, operationalized as comparisons between individuals with convictions and those without. Mental illness and drug use are especially related to convictions by G3 and G2 males. It is important to note that our measures of drug use only reflect self-reported cases of drug use where medical treatment was needed. Drug use in itself is far higher. For example, self-reported drug use of G2 males at age 32 was significantly higher for those with convictions, with 45.3% of convicted persons and 25.0% of non-convicted individuals reporting they had used drugs (*OR* = 1.75, *CI* 1.18–2.61).

There are, however, differences between genders. Convicted females, remarkably. have significantly better health than non-convicted females. This may be because females with convictions are less likely to have physical disabilities prohibiting their engagement in physical activity, had more manual jobs and alcohol was a protective factor against infection, similarly to convicted G2 males at ages 18 and 32 (Shepherd et al., 2002, 2004).

It is possible that females may have worse physical health as their age and criminal career increases, as their antisocial behaviors and drug use take effect physiologically. This is in line with the Ecosocial theory’s explanation of health outcomes and the concept of embodiment, which promotes the idea that human bodies function in the light of social conditions and behaviors that leave biological wear and tear on the body, such as offending over the life course (Krieger, 2005, 2011). Unfortunately, there is a paucity of research in relation to the health of convicted female offenders compared with non-convicted females. No previous studies that we are aware of have investigated the health of convicted females in community samples, so we are unable to compare our results to other research (Moffit et al., 2009).

When comparing G3 males to their G2 fathers at a similar age, we can see broadly similar trends in their health (See Skinner et al., 2020). Table 2 shows that the *OR*s for G2M (health vs offending) are remarkably similar to the *OR*s for G3M. Table 3 shows similarity only for fight injuries.

Similarities in health across generations may not be surprising, as Elder (1998, p. 2) stated: “. . .historical forces shape the social trajectories of family, education, and work, and they in turn influence behavior and particular lines of development,” especially when there are also similar levels of convictions and risk factors for convictions across generations (Farrington, Coid, & Murray, 2009; Farrington et al., 2014, 2015). It is also reasonable to expect that an antisocial developmental history, which is largely influenced by childhood risk factors, such as poor parental supervision, low family income, poor nutrition, parental convictions, and lack of medical care (Farrington et al., 2015; Piquero et al., 2007; Poulton et al., 2002; Ross & Mirowsky, 2001), may culminate in poor midlife health. Antisocial individuals may damage their health by engaging in risky health behaviors (e.g., smoking, drug use, unprotected sex), experience high levels of stress due to conflictual relationships or financial insecurity, or they may have personality traits associated with physiological vulnerability that chronically arouse the physiological fight-or-flight responses in the sympathetic nervous system (Rasmussen et al., 2019). It is likely that these processes, namely the antisocial lifestyles that lead to adverse health outcomes, will worsen with the increasing duration and frequency of criminal careers (Odgers et al., 2007). A partial explanation may also reside in selection factors which drive those with poor health toward crime and imprisonment, although it has been argued that offending and imprisonment themselves create worse health outcomes (Schnittker & John, 2007).

It would be interesting to compare convicted and non-convicted siblings within the same families, controlling for gender, although the number of cases in the CSDD may not be sufficient for detailed analyses. Unfortunately, this is not possible within the scope of the present article. Similarly, it would be interesting to compare the relation between health variables and general versus drug-related offending.

## Implications

It is likely that the antisocial lifestyle of convicted persons causes processes which damage their health over time (Skinner & Farrington, 2021; Skinner et al., 2020). The findings of this study imply that preventing individuals from offending is likely to have substantial benefits for health, and across generations. There needs to be a focus on the interruption of intergenerational patterns of offending (Farrington et al., 2017) and there should be further targeted health promotion interventions that improve the associated health repercussions of offending. Additionally, it is important to maintain a continued focus on programs which address the drug use of male and female offenders, and psychosocial and environmental factors which impact genders differently (Auty et al., 2017). Interventions which focus on cognitive-behavioral treatment programs (Tong & Farrington, 2006, 2008) may be particularly effective in this regard, aiming to reduce reoffending in the parent, but also subsequently to change the criminogenic thinking of offenders which is passed down to their children. Interventions that interrupt the intergenerational transmission of antisocial behavior will pay dividends for individual health and for the economic costs associated with worse health in offender populations.

## Limitations

The CSDD provides a wealth of detailed information about criminal careers and their associations with health, over a 40 year follow up period, with high retention of the original participants. However, the CSDD contains data on mainly British white, working class, inner city individuals born around 1953, so the results may not be generalizable to Asian, Afro-Caribbean, suburban, rural, middle- or upper-class people, or those who spent their childhood in other countries. The G2 males were all boys attending six primary schools in a lower-class area of South London and therefore are only representative of boys in that area at that time. There is also the potential for sampling bias in this study, with a lack of sampling of foster care youth or other system involved youth.

The nature of the measurement of medical data, namely that it was self-reported rather than based on recorded medical data, may have also influenced results. However, a review by Jolliffe and Farrington (2014) showed that self-reports were reliable and valid measures of offending.

This research has focused more on the kinds of activities that are outward expressions of health-related behavior, such as injuries and accidents, and not on more general health problems such as hypertension, heart trouble, and diabetes that are known to impair life functioning and that signal risk for future disease and morbidity. Moreover, this study did not examine the variations that may exist within offenders. Moffitt’s (1993) developmental taxonomy suggests that offenders are not homogeneous. Previously, Life-Course-Persistent, Adolescence-Limited and Late-Onset offenders have been compared in G2 males (Piquero et al., 2007; Skinner et al., 2020). It will be interesting in the future to compare these intergenerational and inter-gender health patterns within these longitudinal offender trajectories.

It was also not possible to contribute directly to, or investigate, the debate on heritability, genetic effects or biosocial theorizations, owing to the lack of genetic data and designs allowing sufficient experimental control of independent variables (Rothman & Greenland, 2005).

Overall, this study provides little information about causal paths from offending to poor health outcomes. Intervention studies using designs that successfully adapt the traditional randomized controlled trial structure are necessary to clarify these. Ideally, prospective longitudinal studies with frequent measures of health and offending are needed, in order to investigate whether changes in health precede, follow or coincide with changes in offending.

## Conclusion

Convicted males continue to show worse health compared to non-convicted persons in the third generation of the CSDD. However, G3 convicted females often report better health than non-convicted females, apart from drug use and road accidents. Our analysis suggests one potential path linking offending to health-related outcomes, that is, through differential involvement in antisocial lifestyles (Bulatao & Anderson, 2004). It is likely that the antisocial lifestyle of offenders causes processes which damage their health over time. There needs to be a focus on the interruption of intergenerational patterns of offending (Farrington et al., 2017) and the associated health repercussions, in addition to a continued focus on programs which address drug use of convicted males and females. Interventions that interrupt the intergenerational transmission of antisocial behavior will pay dividends for individual health and the economic costs associated with worse health in offender populations.
